# Digital health at fifteen: more human (more needed)

**DOI:** 10.1186/s12916-019-1302-0

**Published:** 2019-03-18

**Authors:** Kit Huckvale, C. Jason Wang, Azeem Majeed, Josip Car

**Affiliations:** 1 Black Dog Institute, Hospital Road, Prince of Wales Hospital, University of New South Wales Sydney, Randwick, NSW 2031 Australia; 2 Center for Policy, Outcomes, and Prevention, 117 Encina Commons, Stanford University, Stanford, CA 94305-6019 USA; 3 Department of Primary Care and Public Health, Imperial College London, London, W6 8RP UK; 4 Centre for Population Health Sciences, Lee Kong Chian School of Medicine, Nanyang Technological University, Clinical Sciences Building, 11 Mandalay Road, Singapore, 308232 Singapore

**Keywords:** Digital health, eHealth, Person-centred care, Human factors, Ergonomics, Machine learning

## Abstract

There is growing appreciation that the success of digital health – whether digital tools, digital interventions or technology-based change strategies – is linked to the extent to which human factors are considered throughout design, development and implementation. A shift in focus to individuals as users and consumers of digital health highlights the capacity of the field to respond to secular developments, such as the adoption of person-centred care and consumer health technologies. We argue that this project is not only incomplete, but is fundamentally ‘uncompletable’ in the face of a highly dynamic landscape of both technological and human challenges. These challenges include the effects of consumerist, technology-supported care on care delivery, the rapid growth of digital users in low-income and middle-income countries and the impacts of machine learning. Digital health research will create most value by retaining a clear focus on the role of human factors in maximising health benefit, by helping health systems to anticipate and understand the person-centred effects of technology changes and by advocating strongly for the autonomy, rights and safety of consumers.

## Background

Fifteen years ago, there were no smartphones, modern social media, apps or consumer wearables. The mass deployment of electronic health records, let alone patient-facing digital records, had not yet started. Iterative software development methods were ‘bleeding edge’ and machine learning was only just starting to wake from a 20-year slumber. As *BMC Medicine* went to press for the first time, things that today are taken for granted as core ingredients of digital health – as methods or intervention strategies for evaluation – either did not exist or were contested territory.

It is a mistake to characterise the story of digital health research over the following 15 years as one of simply rapid technological innovation. Digital health has always asserted a translational vision of changed practices and systems of care, enabled by technology, to drive better health outcomes. What has changed, however, is recognition that it is the way in which humans interact with technologies, not abstract properties of technology, that is critical to the success of this vision [1]. This growing focus on ‘human factors’ has underpinned key developments in digital health, spanning intervention development, implementation and the quest for patient-centred care. Yet, as we argue, there is a timely opportunity for a renewed focus on the value of human factors in digital health.

### Digital health@15

The idea that user perspectives should inform the design and evaluation of information systems was not new in 2003, yet its value in healthcare was only starting to be recognised. Tellingly, a comprehensive review of the benefits and challenges of user involvement, published in that year, makes no references to health [2]. It took a decade, first to develop and then to apply, a theoretical understanding of the particular scope for a substantial, human-centred ‘design-reality’ gap [3] in healthcare (where complex, contingent and fast-changing human practices challenge traditional technology design methods). A defining result is a mature understanding of the determinants of failures in large-scale health information technology (IT) implementation programs, such as the UK’s National Programme for IT, and the development of strategies intended to avoid future failures [4].

More recently, the focus on ‘information systems within organisations’ that motivated this earlier work has yielded interest in the roles and contributions of individuals, whether professionals, patients or consumers [1]. This shift can be understood partly in terms of technology developments, such as the development of smartphones and consumer health apps, which place patients and consumers as principal users. Mass adoption and a usage culture of ‘always carried, always on’, creates compelling opportunities for intervention, support and monitoring. However, the development of person-centred care, which seeks to respect the preferences, values and autonomy of individuals, has been equally important as both a guiding philosophy and hallmark of quality in health systems [5].

In some areas, strategic expectations [6] have been realised that digital health acts as enabler of person-centred priorities, such as improved access to care and better-tailored treatment. For example, in mental health, internet-based cognitive behavioural therapy (iCBT) has progressed from proof-of-concept [7] to a well-validated and effective therapy for depression [8], with self-directed approaches offering enhanced convenience and a realistic alternative to pharmacotherapy for mild disease. Notably, the development of iCBT was motivated by human factors-based insights into barriers to traditional face-to-face services, which included stigma and a desire for ‘in the moment’ support for mental distress. The same factors continue to influence contemporary developments, such as the emergence of machine learning-based chatbots that offer tailored support for mental distress. One such service has accrued over 2 million weekly users, who say that they are motivated to use this service because of its convenience and reduced perceptions of stigma [9]. One potential consequence of these highly scalable services – made topical by contemporary staffing shortages in key groups of health professionals, such as primary care physicians and mental health therapists [10] – may be to reduce reliance on face-to-face therapy. Any service delivery benefits, however, need to be weighed carefully against prevalent concerns about the privacy and data governance of these services, clinical safety (for example, handling crisis in mental health apps) and evidence for effectiveness [11]. A 2018 review of reviews of smartphone apps for any health condition identified only 11 ‘prescribable’ products that were backed by randomized controlled trials indicating effectiveness, available to the public and designed for standalone use without clinical intervention [12].

Although the contribution of digital health towards other person-centred care priorities, such as continuity of care, is perhaps less clear cut, it nevertheless continues to underline the importance of a focus on human factors. For example, Denmark is a success story in terms of near-universal adoption of electronic health records [13]. Yet, ostensibly simple changes, such as moving from manual to electronic methods of records transmission, have raised concerns of care fragmentation when information has been lost or delayed because human systems have yet to ‘catch up’. Examples of delayed cancer diagnosis underline the scope for serious effects on health outcomes [13] of these human factors-based failures.

### A person-centred, human factors-based future requires sustained effort

If having a person-centred perspective helps to illuminate the value created by digital health over the past 15 years (as well as highlighting where principal challenges remain), the interface between humans and technology is also key – in our view – to understanding where focus should be directed over the next 15 years.

Firstly, although what unites these examples is evidence for the value of a human factors-based focus (to both shape the success of digital health and, relatedly, probe its failures), this project is substantially incomplete. For digital intervention development, user-focused techniques, such as person-centred design, usability testing and ethnography, are still used infrequently – if at all. Yet this focus is urgently needed to tackle pressing challenges that act as barriers to improved health outcomes, such as poor user engagement with mobile health interventions [14], the capacity of healthcare workforces to take on new digital practices (even if they might alleviate workload in the long-term) when staff burnout is increasing [15], or the continuing tendency to focus on digital health technologies in terms of ‘the product’ rather than its ‘performance’ within care systems [16]. Because the consideration of human factors is an established discipline [17], the opportunity is not around new methods of development per se, but rather around how to effectively draw in and value this expertise as a routine part of digital health development and implementation.

Second is anticipating the effects of a shift in decision-making power towards consumers [5]. From a human factors-based perspective, this is interesting not only because these changes raise questions of access, equity, health literacy and privacy, but because consumerist care that involves greater number of loosely coupled blocks may paradoxically create new challenges for person-centred care not faced by monolithic systems, such as care continuity. Technology-enabled consumer healthcare barely existed even 10 years ago, yet today consumers are increasingly able to select services that operate outside of traditional healthcare structures, work across international borders and even span remote consultation, second opinion, self-management, peer support and healthcare administration. In the USA, where 78% of adults own an app-capable smartphone, by 2015 almost three-fifths had downloaded at least one health app [18]. Today, the market may be dominated by wellness applications. However, positive evaluations of self-management tools and automated diagnostics (as well as rapid developments in the commercial sector) suggest that the future of digital health will lead to the transformation of healthcare structures. A current focus on enabling technical aspects of service integration, such as data standards and interoperability, is necessary and timely, but should not distract from the need to address user factors.

Third is the imminent potential for healthcare delivery in low-income and middle-income countries because over 1 billion people will gain access to smartphones within the next decade. Limited per capita spending on health services means that many will continue to have little or no access to traditional healthcare. Providing health advice via smartphone apps, for example, has the potential to enable access and improve health outcomes in populations on whom the burden of global disease falls disproportionately. Questions of fit with users’ – and providers’ – needs and expectations are as relevant for the success of digital health in these settings as any other [19].

The final development that warrants a continued, strong focus on human factors is the impact of artificial intelligence on systems of care, autonomy and safety. A salient example is the development of interactive ‘bots’ that use machine learning to drive natural language computer interfaces. As these bots become more advanced, there will be new opportunities for interactions between patients and professionals. It is not hard to imagine future three-way scenarios involving patients, professionals and bots. A software bot might, for example, supply empirical insights into long-term condition status and changes based on data collected outside the clinical setting using a patient’s own device. Alternatively, it might provide a safety net for a clinician interpreting imaging or test results. These developments raise important questions concerning the autonomy of patients and medical professionals, as well as impacts on workflows and service utilisation [20]. To meaningfully explore these issues, however, the field must tackle head-on a tendency for computer science-led collaborations that focus only on proof-of-concept scenarios. For publicly funded research that claims a pragmatic focus, there is little utility in developing approaches that may display excellent technical properties, but which are unlikely to be implemented because they do not address fundamental questions of compatibility with working practices, clinical norms or user acceptability.

Machine learning also brings new safety, equity and privacy concerns. The emergence of novel safety risks at the interface between humans and increasingly sophisticated automation in the commercial aviation industry [21] (long held as a model for safety in healthcare) should be a wake-up call as algorithms start to be woven into everything, including discharge planning, enhanced surgical vision and machine learning-based decision support (ML-DSS). New procedural and technical strategies will be needed to detect and address previously unanticipated equity issues, such as poor performance by biased algorithms that learn using data only from specific groups [22]. Changing public attitudes around data governance, spurred on by topical failures in the commercial sector, may directly affect the viability of data-hungry applications, such as digital phenotyping efforts that seek to build new risk-prediction models using behavioural insights gathered automatically from smartphones and wearables [23]. These applications have genuine potential to improve patient-centred health outcomes and experience, for example, by allowing individuals at risk of relapse in mental health to detect early signs without the burden of manual self-monitoring, but only if they are acceptable to users.

## Conclusions

The injunction that ‘health IT should not be viewed as an end in itself’ [6] has never been more appropriate. The potential for a rich understanding of the interactions between technology and its users to help identify and address barriers to desired health outcomes offers a clear agenda for digital health research. It also provides a warrant for the value of this research domain at a time when the ubiquity of information technology might otherwise make digital technologies ‘just another’ delivery mechanism for intervention or systems change. What should happen when ‘partnership models’ of decision-making that combine professionals, consumers and software agents are shown to drive superior outcomes and reduced clinical risk? Or when positive experiences with sophisticated software agents in commercial settings lead consumers to expect the same kind of convenience in healthcare?

Digital health research will create most value by retaining a clear focus on the role of human factors in maximising health benefit, by helping health systems to anticipate and understand the person-centred effects of technology changes and by advocating strongly for the autonomy, rights and safety of consumers in a healthcare landscape in which technology will play an ever-greater role.

## References

[1] Granja C, Janssen W, Johansen MA (2018). Factors determining the success and failure of eHealth interventions: systematic review of the literature. J Med Internet Res.

[2] Kujala S (2003). User involvement: a review of the benefits and challenges. Behav Inform Tech.

[3] Heeks R (2006). Health information systems: failure, success and improvisation. Int J Med Inform.

[4] Greenhalgh T, Wherton J, Papoutsi C, Lynch J, Hughes G, A’Court C (2017). Beyond adoption: a new framework for theorizing and evaluating nonadoption, abandonment, and challenges to the scale-up, spread, and sustainability of health and care technologies. J Med Internet Res.

[5] Berwick D (2009). What ‘patient-centered’ should mean: confessions of an extremist. Health Aff.

[6] Epstein RM, Fiscella K, Lesser CS, Stange KC (2010). Why the nation needs a policy push on patient-centered health care. Health Aff.

[7] Christensen H, Griffiths KM (2002). The prevention of depression using the internet. Med J Aust.

[8] Karyotaki E, Riper H, Twisk J, Hoogendoorn A, Kleiboer A, Mira A (2017). Efficacy of self-guided internet-based cognitive behavioral therapy in the treatment of depressive symptoms: a meta-analysis of individual participant data. JAMA Psychiatry.

[9] Nutt AE. ‘The Woebot will see you now’ — the rise of chatbot therapy: Washington Post; 2017. https://www.washingtonpost.com/news/to-your-health/wp/2017/12/03/the-woebot-will-see-you-now-the-rise-of-chatbot-therapy. Accessed 04 Mar 2019

[10] Majeed A (2017). Shortage of general practitioners in the NHS. BMJ..

[11] Torous J (2016). Mobile telephone apps first need data security and efficacy. BJPsych Bull.

[12] Byambasuren O, Sanders S, Beller E, Glasziou P (2018). Prescribable mHealth apps identified from an overview of systematic reviews. NPJ Digital Med.

[13] Kierkegaard P (2013). eHealth in Denmark: a case study. J Med Syst.

[14] Torous J, Nicholas J, Larsen ME, Firth J, Christensen H (2018). Clinical review of user engagement with mental health smartphone apps: evidence, theory and improvements. Evid Based Ment Health.

[15] Dyrbye LN, Shanafelt TD, Sinsky CA, Cipriano PF, Bhatt J, Ommaya A (2017). Burnout among health care professionals: a call to explore and address this underrecognized threat to safe, high-quality care. NAM perspectives.

[16] Greenhalgh T, Procter R, Wherton J, Sugarhood P, Hinder S, Rouncefield M (2015). What is quality in assisted living technology? The ARCHIE framework for effective telehealth and telecare services. BMC Med.

[17] Russ AL, Fairbanks RJ, Karsh B-T, Militello LG, Saleem JJ, Wears RL (2013). The science of human factors: separating fact from fiction. BMJ Qual Saf.

[18] Krebs P, Duncan DT (2015). Health app use among us mobile phone owners: a national survey. JMIR Mhealth Uhealth.

[19] Opoku D, Stephani V, Quentin W (2017). A realist review of mobile phone-based health interventions for non-communicable disease management in sub-Saharan Africa. BMC Med.

[20] Cabitza F, Rasoini R, Gensini G (2017). Unintended consequences of machine learning in medicine. JAMA..

[21] Rankin A, Woltjer R, Field J (2016). Sensemaking following surprise in the cockpit—a re-framing problem. Cogn Technol Work.

[22] Gianfrancesco MA, Tamang S, Yazdany J, Schmajuk G (2018). Potential biases in machine learning algorithms using electronic health record data. JAMA Intern Med.

[23] Onnela JP, Rauch SL (2016). Harnessing smartphone-based digital phenotyping to enhance behavioral and mental health. Neuropsychopharmacology..

